# Unconventional Secretion of Angiogenic Sonic Hedgehog–Containing Extra‐Large Extracellular Vesicles is Driven by PI3K–Rab18‐GDP Signalling

**DOI:** 10.1002/jex2.70112

**Published:** 2026-01-28

**Authors:** Shuo Wang, Rio Imai, Yuya Kaneko, Yosuke Tanaka

**Affiliations:** ^1^ Laboratory of Molecular Cell Dynamics/Cytoarchitectonics, Department of Cell Biology and Anatomy, Graduate School of Medicine The University of Tokyo Hongo Tokyo Japan

**Keywords:** angiogenesis, extra‐large extracellular vesicles (XLEVs), heat shock protein 90α (Hsp90α), neutral sphingomyelinase 2 (nSMase2), PI3K signalling, Rab18‐GDP, sonic hedgehog (SHH)

## Abstract

Extra‐large extracellular vesicles (XLEVs), with diameters > 600 nm, are increasingly recognised as mediators of specialized modes of intercellular communication; however, the molecular mechanisms governing their biogenesis and functional regulation remain poorly understood. Here, we show that PI3K–Rab18‐GDP signalling promotes the secretion of XLEVs from human mesenchymal stem cells (hMSCs) and fibroblasts. These vesicles are highly enriched in sonic hedgehog (SHH) and display potent pro‐angiogenic activity. We further demonstrate that Rab18 functions as a key regulator of this pathway specifically in its GDP‐bound form, which can be enriched by the Rab inhibitor CID1067700 or by pharmacological activation of PI3K using SF1670. Rab18‐GDP preferentially accumulates in the perinuclear region, where it promotes the formation of SHH‐XLEV precursors from endosomal compartments. Mechanistically, PI3K–Rab18‐GDP signalling recruits heat shock protein 90α (Hsp90α) and neutral sphingomyelinase 2 (nSMase2), facilitating polarized release of SHH‐XLEVs from the perinuclear–plasma membrane interface, accompanied by an Hsp90α‐enriched extracellular assembly. Together, these findings identify a PI3K–Rab18‐GDP–dependent secretory pathway for SHH‐XLEVs and provide a framework for understanding how XLEV biogenesis is coupled to SHH‐associated angiogenic signalling in developmental and regenerative contexts.

## Graphical abstract

Human mesenchymal stem cells conditionally secrete sonic hedgehog (SHH)‐containing extra‐large extracellular vesicles (XLEVs) upon PI3K activation, which enriches the GDP‐bound form of Rab18. Rab18‐GDP drives perinuclear maturation and polarized vertical release of SHH‐XLEVs via Hsp90α‐ and nSMase2‐dependent mechanisms. These angiogenic XLEVs robustly stimulate endothelial network formation, revealing an unconventional secretory pathway linking PI3K signaling to regenerative angiogenesis.

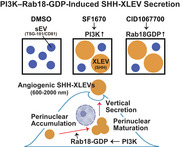

## 1 Introduction

Extracellular vesicles (EVs) mediate intercellular communication by transferring proteins, lipids, and nucleic acids between cells and tissues, thereby modulating the functional states of recipient cells (Abels and Breakefield 2016; Buzas 2023; Veziroglu and Mias 2020). In multicellular organisms, this mode of communication contributes not only to tissue homeostasis but also to the establishment and maintenance of morphogen gradients during development (Tanaka et al. 2023; Teleman et al. 2001; Wang et al. 2022; Wu et al. 2022). EVs derived from human mesenchymal stem cells (hMSCs) have attracted particular interest because of their regenerative potential and their ability to reduce risks associated with allogeneic cell transplantation, including infection and immune rejection (Musiał‐Wysocka et al. 2019; Nakazaki et al. 2021; Nakazaki et al. 2024; Zhang et al. 2020). Notably, loading of the morphogen sonic hedgehog (SHH) into hMSC‐derived EVs has been shown to enhance their therapeutic efficacy in ischemic heart failure models (Mackie et al. 2012). Despite these advances, the cellular mechanisms governing the formation and regulated secretion of this clinically relevant EV subclass remain incompletely understood.

We previously identified a PI3K‐induced pathway that promotes the biogenesis of SHH‐containing extra‐large EVs (SHH‐XLEVs) through molecular and cellular analyses of *Kif3b^LacZ/LacZ^* polydactyly mouse embryos (Wang et al. 2022). In the developing limb bud, a distal‐to‐proximal FGF/PI3K signalling gradient within the mesenchyme selectively enhances SHH‐XLEV secretion and transcytosis in the peripheral layer. This spatially restricted secretion behaviour may underlie the formation of a sustained SHH gradient required for proper digit patterning. Importantly, PI3K‐dependent SHH‐XLEV secretion could be recapitulated in SHHN‐EGFP–expressing fibroblasts, where exceptionally large fluorescent vesicular structures were released from cells, consistent with a subclass of extra‐large EVs (XLEVs). SHH‐XLEV precursor organelles were found to associate with a set of previously described “ART‐EV” markers, including Axl, Rab18, and Tmed10 (Coulter et al. 2018; Wang et al. 2022). However, the biological potency and mechanistic basis of SHH‐XLEV biogenesis have remained largely unexplored.

Rab18 is a member of the Rab GTPase superfamily, which orchestrates membrane trafficking and organelle dynamics within the cytoplasm (Schäfer et al. 2000). Rab proteins cycle between GTP‐ and GDP‐bound conformations, each engaging distinct sets of effector proteins and thereby conferring directionality and specificity to intracellular transport processes (Homma et al. 2021; Klopper et al. 2012). Differential interactions of GTP‐ and GDP‐bound Rab proteins with plus‐ and minus‐end–directed molecular motors can result in either dispersal or perinuclear accumulation of Rab‐associated organelles (Niwa et al. 2008; Ueno et al. 2011; Wanschers et al. 2008). Rab18 is known to interact with kinectin, a binding partner of the kinesin‐1 light chain on the endoplasmic reticulum (Guadagno et al. 2020). Given that kinesin‐1 plays a central role in organelle distribution and ER organization (Tanaka et al. 2024; Tanaka et al. 1998), the nucleotide‐state–dependent interaction between Rab18 and kinesin‐1 is well positioned to influence the spatial dynamics of SHH‐XLEV precursor organelles.

Heat shock protein 90α (Hsp90α) is a multifunctional molecular chaperone with emerging roles at the interface of protein folding, membrane dynamics, and extracellular signalling. In addition to forming cytoplasmic condensates with kinesin‐1 and supporting ER‐associated protein folding and calcium channel homeostasis (Tanaka et al. 2024), Hsp90α has been implicated in membrane deformation during exosome biogenesis (Lauwers et al. 2018). Extracellularly, Hsp90α can stabilize secreted enzymes, promote metastatic niche formation, and modulate autoimmune responses (Baker‐Williams et al. 2019; Moin et al. 2020). These diverse activities raise the possibility that Hsp90α may participate in unconventional EV secretion pathways that couple intracellular trafficking to extracellular signalling functions.

In the present study, we investigated whether pharmacological modulation of Rab activity using the Rab inhibitor CID1067700 affects the secretion of small EVs (sEVs) or XLEVs. We found that CID selectively and robustly enhances the secretion of SHH‐XLEVs, while exerting distinct effects on sEV release. We further demonstrate that SHH‐XLEVs possess strong pro‐angiogenic activity and delineate a PI3K–Rab18‐GDP–dependent mechanism underlying their biogenesis. PI3K signalling enriches the GDP‐bound form of Rab18, which accumulates in perinuclear endosomal compartments following dissociation from kinesin‐1. These compartments give rise to SHH‐XLEV precursors through the recruitment and activation of Rab18‐GDP effector proteins, including Hsp90α and neutral sphingomyelinase 2 (nSMase2). Upon stimulation, SHH‐XLEVs are preferentially released from the perinuclear region, and prolonged activation results in the appearance of Hsp90α‐enriched extracellular structures associated with these vesicles. Together, our findings establish a mechanistic framework for Rab18‐GDP–mediated SHH‐XLEV secretion and provide insight into the cell biology of unconventional XLEV release, with implications for angiogenic signalling and regenerative contexts.

## 2 Materials and Methods

### 2.1 Cells

Human mesenchymal stem cells (hMSCs) derived from bone marrow were purchased from Lonza (Cat. No. PT‐2501) and cultured according to the manufacturer's instructions. Briefly, hMSCs were maintained in MSCBM Mesenchymal stem cell basal medium (PT3238, Lonza) supplemented with MSCGM SingleQuots supplements and growth factors (PT4105, Lonza) in 10‐cm dishes (#150466, Thermo Scientific). Cells were detached using trypsin/EDTA (CC‐3232, Lonza) for 5 min at 37°C, passaged every 7 days at 5000–6000 cells/cm^2^, and used within six passages. Culture medium was replaced every 3 days.

Immortalized hTERT‐MSCs (UE7T‐13) (Mori et al. 2005) were obtained from the JCRB Cell Bank (Osaka, Japan; Cat. No. JCRB1154) and maintained in DMEM (FUJIFILM Wako, Japan) supplemented with 10% fetal bovine serum, 2 mM l‐glutamine (Invitrogen), and penicillin–streptomycin (1:100; Invitrogen). For secretome collection, cells were treated with the indicated compounds for 2 h in EV‐Up medium (FUJIFILM Wako).

Human umbilical vein endothelial cells (HUVECs) were purchased from Lonza (C‐2519AS) and cultured in EBM‐2 Endothelial Cell Growth Basal Medium‐2 (CC‐3156, Lonza) supplemented with EGM‐2 SingleQuots (CC‐4176, Lonza), following the manufacturer's protocol. For passaging, cells were rinsed with HEPES buffer (CC‐5022, Lonza), detached with trypsin/EDTA (CC‐5012, Lonza) for 3–5 min at 37°C, neutralized with trypsin neutralizing solution (CC‐5002, Lonza), and reseeded at 2,500 cells/cm^2^. HUVECs were used within four passages. Culture medium was replaced every 3 days.

NIH3T3 cells were cultured in high‐glucose DMEM containing 10% FBS (F7524, JRH Biosciences) under standard conditions. For stable expression of EGFP‐Rab18 or *piggybac‐SHHN‐StayGold* constructs, cells were transfected using Lipofectamine LTX with PLUS reagent (#15338100, Thermo Fisher) or a Neon electroporator (Invitrogen) and selected with either G418 (200 µg/mL; #10131035, Thermo Fisher) or puromycin (15 µg/mL; P8833, Sigma‐Aldrich), followed by subcloning for cell biological analyses.

### 2.2 Pharmacology

CID1067700 (Sigma‐Aldrich, SML0545) was dissolved at 40 mM in DMSO and used at 40 µM (typically overnight). TAS‐116 (MedChemExpress, HY‐15785) was dissolved at 1 mM in DMSO and used at 0.5 µM for 48 h. GW4869 (Cayman Chemical, #13127) was dissolved at 5 mM in DMSO and used at 1.25 µM (typically overnight). SF1670 (Selleck, S7310) was used at 250 nM as described previously (Tanaka et al. 2016). ZSTK474 (Selleck, S1072) was dissolved at 300 µM in DMSO and applied for 1 h at 1 µM. LY294002 (Cayman Chemical, #70920) was dissolved at 50 mM in DMSO and applied for 8 h at 50 µM.

### 2.3 EV Secretion and Preparation of Secretomes

EV handling was performed in accordance with the MISEV2023 recommendations (Welsh et al. 2024). hMSCs were cultured in 6‐well plates or 10‐cm dishes at 5000–6000 cells/cm^2^ for 5–6 days and then treated with SF1670 (250 nM), CID1067700 (40 µM), or DMSO (1:1,000) in EV‐Up medium (#053‐09451, FUJIFILM Wako) for 1–24 h. Conditioned media were collected and centrifuged at 200–800 × *g* for 10–30 min at 4°C to remove cells and debris. When indicated, samples were concentrated to 1 mL using a 30 kDa MWCO centrifugal filter (Amicon Ultra, 30 kDa).

For precipitation‐based concentration, conditioned media were mixed with 1/2 volume of total exosome isolation Reagent (Thermo Fisher), incubated overnight at 4°C with rotation, and centrifuged at 10,000 × *g* for 60 min at 4°C (bench‐top centrifuge; TOMY Seiko). Alternatively, cleared media were subjected directly to differential centrifugation at 17,900 × *g* for 2 h at 4°C, followed by washing the pellet with PBS. Pellets were resuspended in PBS or PBS‐HAT medium (Görgens et al. 2022), stored at 4°C, and used within 2 weeks.

### 2.4 Antibodies

Primary antibodies were as follows: rabbit anti‐SHH (20697‐1‐AP, RRID: AB_10694828, 1:300), rabbit anti‐CD63 (25682‐1‐AP, RRID: AB_2783831, 1:500), and rabbit anti‐calnexin‐1 (10427‐2‐AP, RRID: AB_2069033, 1:500) (Proteintech); rat anti‐Hsp90α clone 16F1 (ADI‐SPA‐835, RRID: AB_311881, 1:300) (Enzo Life Sciences); mouse anti‐TSG101 clone 4A10 (MA1‐23296, RRID: AB_2208088, 1:300) (Thermo Fisher); rabbit anti‐CD81 D5O2Q (10037, RRID: AB_2714207, 1:300) (Cell Signalling Technology); rabbit anti‐GST (A5838, RRID: AB_258261, 1:300), mouse anti‐γ‐adaptin/AP‐1 (A4200, RRID: AB_476720, 1:300), mouse anti‐AP‐3 (A4825, RRID: AB_258203, 1:300), and mouse IgM anti‐ceramide (C8104, RRID: AB_259087, 1:300) (Sigma‐Aldrich). Rabbit anti‐KIF5A N‐terminal antibody (RRID: AB_2571744, 1:1000) was described previously (Kanai et al. 2000).

Secondary antibodies included HRP‐conjugated anti‐rabbit and anti‐mouse IgG (1:1000; GE Healthcare Life Sciences), alkaline phosphatase–conjugated anti‐rabbit and anti‐mouse IgG (1:1000; Cappel), and Alexa Fluor 405/488/568–conjugated secondary antibodies (anti‐mouse, anti‐rat, anti‐rabbit IgG and anti‐mouse IgM; 1:200–1:500; Thermo Fisher).

### 2.5 Expression Vectors

SHHN‐EGFP expression vectors (plasmid and adenovirus) encoding amino acids 1–198 of mouse SHH‐N were described previously (Wang et al. 2022). To generate SHHN‐tagRFP expression vector, Shh‐N was amplified using Sal‐SHHATGKozak‐fwd (5′‐ACCGTCGACCATGGTGCTGCTGCTGGCCAGATG‐3′) and Bam‐SHH198‐rev (5′‐CAAGGATCCCGGCCGCCGGATTTGGCCGCCACG‐3′), and ligated into *pTagRFP‐N* (FP142, Evrogen) to yield *pSHHN‐tagRFP*.

To construct Hsp90α‐tagRFP expression vector, mouse Hsp90aa1 cDNA (FANTOM clone I1C0020P08) (Kawai et al., 2001) was amplified using Xho+hsp90aa1atgFw (5′‐ACCCTCGAGCTATGCCTGAGGAAACCCAGACCCAAG‐3′) and Kpn+hsp90aa1stopRev (5′‐ACCGGTACCTTAGTCTACTTCTTCCATGCGTGATGTGTC‐3′), and ligated into *pTagRFP‐C1* at *XhoI/KpnI* sites. Expression units were transferred to the ViraPower Adenoviral Expression System (Thermo Fisher) according to the manufacturer's instructions.

EGFP‐Rab18 (Addgene #49550; RRID: Addgene_49550), EGFP‐Rab18Q67L (Addgene #49596; RRID: Addgene_49596), and EGFP‐Rab18S22N (Addgene #49597; RRID: Addgene_49597) were gifts from Marci Scidmore (Huang et al. 2010). For bacterial expression of GST‐Rab18 proteins, Rab18 inserts were amplified using EcoRI+Rab18‐F (5′‐TCCGAATTCCATGGACGAGGACGTGCTGACCACTCTG‐3′) and Not+Rab18‐R (5′‐AATGCGGCCGCCTATAGCACAGAGCAGTAACCGCCGCAGGCG‐3′), and cloned into *pGEX‐4T‐3* (Cytiva Life Sciences) at *EcoRI/NotI* sites.

The *piggyBac‐SHHN‐mStayGold* expression vector was constructed by Yiming Li. Briefly, *Shh‐N* cDNA was amplified from *pSHHN‐EGFP* (Wang et al. 2022) using newPiggymShhN‐F (5′‐TCAGATCCGCTAGCGCCACCATGGTGCTGCTGCTGGCCAG‐3′) and newPiggymShhN‐R (5′‐ACCATTGATCCGCCGCCACCGCCGCCGGATTTGGCCGC‐3′). The *pPB[Exp]‐Puro* backbone (VectorBuilder, VB240319‐1202jdr), CMV promoter (from *pEGFP‐N1*), and *mStayGold* cDNA (Hirano et al., 2022; provided by Atsushi Miyawaki, RIKEN) were amplified using the primer sets listed in the original manuscripts and assembled using Gibson Assembly (E2611, NEB) at 50°C for 60 min. DH5α transformation, colony screening, plasmid preparation, and Sanger sequencing were performed using standard procedures. The hyPBase expression vector *pRP[Exp]‐mCherry‐CAG>hyPBase* was purchased from VectorBuilder (Cat. No. pDNA(VB010000‐9365tax)‐P) for generating *piggyBac‐SHHN‐StayGold* stably expressing cells.

### 2.6 Transfection and Transduction

Plasmids were introduced using Lipofectamine LTX with PLUS reagent (Thermo Fisher), and adenoviral transduction was performed using the ViraPower system (Thermo Fisher), following the manufacturers’ protocols.

### 2.7 Nanoparticle Tracking Analysis (NTA)

Conditioned media were cleared by centrifugation, diluted 1:50 in PBS, and analyzed using a ZetaView instrument (Particle Metrix) in “highest” mode. Data were processed using Prism (V7 and V9.3.1; GraphPad).

### 2.8 Flow Cytometry (FACS) Analysis of Conditioned Media

NIH3T3 cells stably expressing piggyBac‐SHHN‐mStayGold were stimulated with CID (40 µM) for 1 h. Conditioned media were cleared by centrifugation and analyzed directly on a CytoFLEX flow cytometer (Beckman Coulter; Cat. No. B53018). Particle fluorescence (excitation 488 nm; emission 525 nm) was plotted against side scatter (SSC‐H). Polystyrene beads (300, 600, and 1000 nm) were used as size references. Due to differences in refractive index between beads and EVs, SSC‐H–based size estimates were interpreted as approximate. The gate for large fluorescent particles (P1; Figure 2K–M) was applied and compared between transfected and nontransfected cells.

### 2.9 Transmission Electron Microscopy (TEM)

TEM was performed as described previously (Nonaka et al. 1998). EV pellets were fixed in half‐Karnovsky fixative (2% paraformaldehyde, 2.5% glutaraldehyde in 0.1 M cacodylate buffer, pH 7.4) at 37°C for 10 min and then at 4°C overnight, post‐fixed with 1% OsO_4_ in 0.1 M cacodylate buffer on ice for 1.5 h, stained with 1% uranyl acetate for 1 h, dehydrated through a graded ethanol series, and embedded in Quetol‐812 (Nisshin EM). Ultrathin sections were stained with uranyl acetate and lead citrate and imaged using a JEM‐2000 (100 keV) and/or JEM‐1400 Flash microscope (80 or 100 keV) (JEOL).

### 2.10 In Vitro Angiogenesis Assay

Tube formation assays were performed using a protocol based on Lonza recommendations (Arnaoutova and Kleinman 2010). Briefly, eight‐well glass‐bottom chambers (#155409, Thermo Scientific) or 15‐well μ‐Slides (ib81506, Ibidi) were coated with Matrigel (#356231, Corning; 250 µL/well) and incubated at 20°C for 10 min, followed by polymerization at 37°C for ≥ 30 min in 5% CO_2_.

For assays, HUVECs were seeded at 4.3–8 × 10^4^ cells/cm^2^ and mixed with total EV fractions isolated from hMSC‐conditioned media at approximately 10 µg/mL EV protein concentration. Where indicated, HUVECs were transfected with *piggyBac‐SHHN‐StayGold* using a Neon electroporator (Thermo Fisher) and cultured for 3 days prior to the assay. After 90 min, samples were imaged using a ZEISS LSM780 (Plan‐Apochromat 10×/0.45 M27; DIC mode) or a Nikon A1R microscope at 37°C in 5% CO_2_. Quantification was performed using ImageJ (V1.53q) (Schindelin et al. 2015), scoring tube number (Fujio et al. 2017; Lin et al. 2019).

### 2.11 Immunoblotting

Immunoblotting was performed as described previously (Tanaka et al. 2016). EV preparations were lysed in 1× RIPA buffer, mixed with 4× Laemmli sample buffer (1/3 volume), boiled at 98°C for 5 min, separated by SDS‐PAGE, and transferred to PVDF membranes (Immobilon‐P, EMD Millipore). Membranes were incubated with primary and HRP‐conjugated secondary antibodies using Can Get Signal Solutions 1 and 2 (TOYOBO) and developed by ECL (GE Healthcare Life Sciences). Signals were acquired using an ImageQuant LAS 4000 mini system (GE Healthcare Life Sciences) and quantified with ImageJ. Alternatively, alkaline phosphatase–conjugated secondary antibodies (Cappel) were used with BCIP/NBT (Roche) (Kondo et al., 1994). Molecular weight markers were from Bio‐Rad.

### 2.12 GST Pull–Down Assay

GST pull–down assays were performed as described previously (Niwa et al. 2008; Xu et al. 2018), using B‐PER Bacterial Cell Lysis Reagent (Thermo Fisher Scientific) for bacterial lysate preparation.

### 2.13 OptiPrep Density Gradient Centrifugation

Iodixanol (OptiPrep) density gradient centrifugation was performed as described (Kowal et al. 2016). CID‐stimulated conditioned media were cleared at 800 × *g* for 10 min, and EVs were pelleted at 10,000 × *g* for 60 min. Pellets were resuspended in 3 mL of 30% OptiPrep in 250 mM sucrose, 10 mM Tris (pH 8.0), and 1 mM EDTA (pH 7.4). Gradients were prepared by overlaying 0.9 mL of 20%, 0.8 mL of 10%, and 0.8 mL of 0% OptiPrep solutions. Samples were centrifuged at 350,000 × *g* for 60 min at 4°C (SW55Ti rotor; Optima XL‐100K, Beckman Coulter). Fractions (500 µL) were collected, diluted with 1 mL PBS, and pelleted at 100,000 × *g* for 30 min at 4°C prior to immunoblotting.

### 2.14 Immunofluorescence Microscopy

Cells were plated in 35‐mm glass‐bottom dishes (D11130H, Matsunami) precoated with poly‐l‐lysine (P4707, Sigma; 1:1,000) for 1 h. Cells were fixed with either 2% paraformaldehyde/0.1% glutaraldehyde in PBS or 4% paraformaldehyde in PBS at 37°C for 10 min. Where indicated, cells were permeabilized with 0.1% Triton X‐100 in PBS for 5 min at 20°C, incubated with primary antibodies overnight at 4°C, and then with secondary antibodies for 1 h at 20°C, followed by three PBS washes. Imaging was performed using a spinning disk confocal microscope (Yokogawa/ZEISS), a ZEISS LSM780 microscope with Airyscan, or a Nikon A1R confocal microscope. Three‐dimensional reconstructions were generated using Imaris 8 (Bitplane) or Fiji/ImageJ (Schneider et al. 2012). Line profiles and fluorescence intensities were quantified in Fiji/ImageJ.

### 2.15 Live Imaging of SHH‐XLEVs in Culture Medium

Live imaging was performed essentially as described (Wang et al. 2022). For quantification of extracellular SHH‐XLEV density (Figure 2A–F), NIH3T3 cells were plated at 1 × 10^4^ cells/well in LabTek II Chambered #1.5 coverglass chambers precoated with poly‐l‐lysine. Cells were transduced with adenoviral SHHN‐EGFP on DIV3, switched to complete medium with or without compounds on DIV4, and imaged 24 h later using a spinning disk microscope at 5 s intervals at 37°C in 5% CO_2_ (Tanaka et al. 2016). Fluorescent particles exhibiting Brownian motion were tracked and quantified using Imaris 8.

### 2.16 Live Imaging of XLEV Biogenesis

For live imaging of XLEV biogenesis, cells were transduced or transfected with fluorescently tagged constructs as indicated. EGFP‐Rab18 (WT/S22N/Q67L) constructs were introduced using Lipofectamine LTX and selected with G418 (200 µg/mL). SHHN‐tagRFP was introduced by Lipofectamine LTX, and Hsp90α‐tagRFP and SHHN‐EGFP were introduced by adenoviral transduction. The *piggyBac‐SHHN‐mStayGold* construct was introduced together with the PBase expression vector using a Neon electroporator (Invitrogen/Thermo Fisher). After pharmacological treatments, imaging was performed using a CSU‐W1 spinning disk system (Yokogawa/ZEISS), an LSM780‐Airyscan microscope (ZEISS), or a ZEISS Lattice Light Sheet 7 microscope. For long‐term imaging on spinning disk and LSM780 systems, cells were maintained in humidified chambers at 37°C with 5% CO_2_.

### 2.17 Statistical Analysis

Quantification methods are described in the relevant sections. Statistical analyses were performed using Excel for Microsoft 365 and Prism (V7 and V9.3.1; GraphPad), including one‐sided Welch's *t* tests, one‐way ANOVA, two‐way ANOVA, and chi‐square tests. For peripheral distribution score (PDS; Figure 4C,F), fluorescence intensity within 2 µm from the cell periphery was divided by total cytoplasmic fluorescence intensity.

### 2.18 Study Approval

Use of hMSCs was approved by the Graduate School of Medicine, The University of Tokyo (approval No. 2023383NI). Recombinant DNA experiments were approved by the Graduate School of Medicine, The University of Tokyo (approval No. G24M0427). C57BL/6J mice were purchased from CLEA Japan and maintained under specific pathogen‐free conditions with approval from The University of Tokyo, Graduate School of Medicine (protocol No. A2024M050).

## 3 Results

### 3.1 The Rab Antagonist CID1067700 Induces SHH‐Rich XLEV Secretion

**FIGURE 1:**
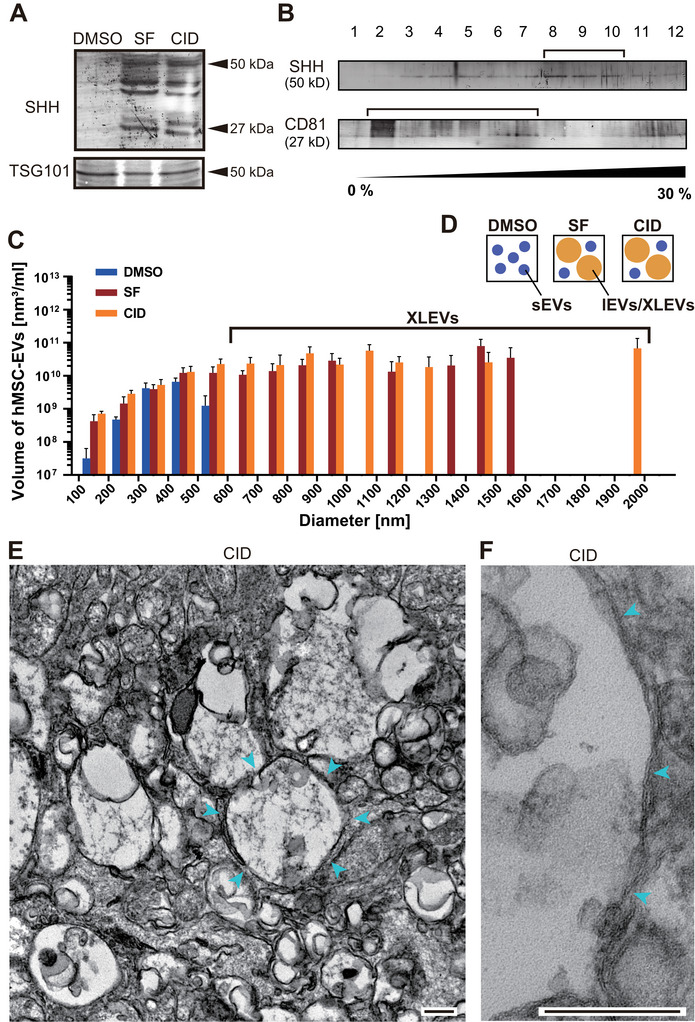
SF1670 (SF) and CID1067700 (CID) promote secretion of sonic hedgehog–containing extra‐large extracellular vesicles (SHH‐XLEVs) from human mesenchymal stem cells (hMSCs). (A) Immunoblot analysis of EVs isolated from hMSC‐conditioned media following overnight treatment with 0.1% dimethyl sulfoxide (DMSO), 250 nM SF, or 40 µM CID, probed with the indicated antibodies. Related to Figure S1. (B) OptiPrep (0%–30%) density gradient fractionation of secretomes from CID‐treated hMSCs (40 µM, 24 h), immunoblotted for SHH and CD81. SHH and CD81 were predominantly detected in fractions 8–10 and 2–7, respectively. (C) Nanoparticle tracking analysis (NTA) of EVs from bone‐marrow‐hMSC‐conditioned media following the indicated treatments (three independent experiments). SF and CID selectively promoted secretion of XLEVs larger than 600 nm (Bracket). Error bars represent mean ± SEM; *n* = 3. (D) Schematic illustration of secretomes containing small EVs (sEVs) and/or extra‐large EVs (XLEVs). (E, F) Transmission electron microscopy of 17,900 × *g* EV pellets from CID‐treated hTERT‐MSC‐conditioned media (40 µM, 2 h). Arrowheads indicate XLEVs. Scale bars, 200 nm. Related to Figure S2.

Bone marrow–derived hMSCs were stimulated overnight with DMSO (vehicle control), the PI3K agonist SF1670 (SF), or the Rab antagonist CID1067700 (CID). Secretomes were collected from pre‐cleared conditioned media and analyzed by immunoblotting. The small EV marker TSG101 was detected in secretomes from all treatment conditions (Figure 1A). In contrast, SHH protein was detected exclusively in secretomes derived from SF‐ and CID‐treated cells, but not from DMSO‐treated controls. This PI3K dependency of SHH‐containing EV secretion is consistent with our previous observations in a polydactyly mouse model (Wang et al. 2022).

Short‐term CID treatment (2 h) of hTERT‐MSCs similarly induced the secretion of SHH‐containing EVs, while reducing the levels of canonical sEV markers (TSG101 and CD63) and ER‐derived contaminants (Figure S1). These findings suggested that CID preferentially enhances the secretion of a distinct SHH‐rich EV population.

To compare the physical properties of these vesicles, CID‐induced secretomes were subjected to density gradient centrifugation. SHH‐positive vesicles were detected in denser fractions than conventional CD81‐positive sEVs (Figure 1B). Nanoparticle tracking analysis (NTA) further revealed that EVs larger than 600 nm were detected exclusively in SF‐ and CID‐treated secretomes (SF, 3/3; CID, 3/3), but not in DMSO controls (0/3; *p* = 0.01, chi‐square test; Figure 1C). In contrast, EVs smaller than 600 nm were present in all conditions. Together, these results indicate that SF and CID selectively promote the secretion of extra‐large EVs (XLEVs), supporting the existence of a secretion mechanism distinct from that governing sEV release (Figure 1D).

Transmission electron microscopy (TEM) of EV pellets revealed a marked increase in electron‐lucent vesicular structures exceeding 600 nm in CID‐treated samples compared with DMSO controls (Figure 1E; Figure S2A). These structures were frequently enclosed by lipid bilayers, consistent with their identity as EVs rather than protein aggregates or lipoprotein particles (Figure 1F; Figure S2B,C). Quantitative size analysis confirmed a significant increase in XLEV abundance following CID treatment (Figure S2D,E), in agreement with NTA measurements.

### 3.2 CID Facilitates SHH‐XLEV Biogenesis Through Enrichment of Rab18‐GDP

**FIGURE 2:**
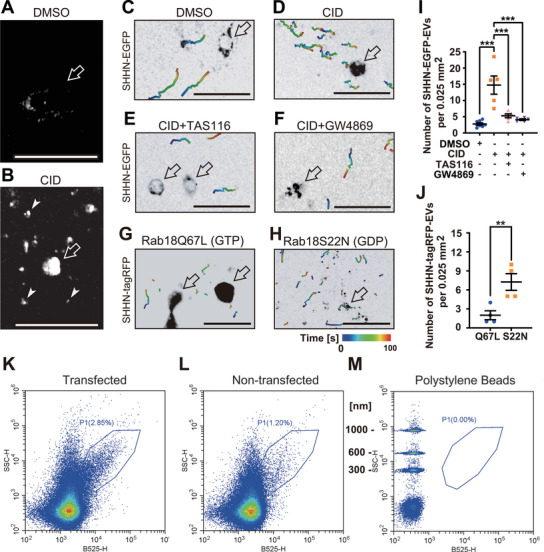
CID1067700 (CID) promotes secretion of SHH‐XLEVs. (A–J) Spinning disk confocal microscopy of SHH‐XLEVs produced by SHHN‐EGFP–transduced (A–F) or SHHN‐tagRFP– and Rab18 mutant–EGFP–transduced NIH3T3 cells (G, H) following treatment with DMSO (C), CID (40 µM, 24 h; D), CID + TAS‐116 (0.5 µM, 48 h; E), or CID + GW4869 (1.25 µM, 24 h; F).(A, B) Single‐frame images and (C–H) particle tracking images are shown, (I, J) with quantification. Open arrows indicate transduced cells; arrowheads indicate XLEVs. Scale bars, 50 µm. Colour bar indicates time (s). SHHN transduction efficiency was approximately 100%. **p < 0.01; ***p < 0.001; one‐way ANOVA (I; *n* = 5–8) and Welch's *t* test (J; *n* = 4). Related to Movies S1 and S2. (K–M) Flow cytometry analysis of conditioned media from SHHN‐mStayGold–expressing (K) and nonexpressing (L) NIH3T3 cells treated with CID (40 µM, 1 h), compared with polystyrene beads of defined sizes (M). The *x*‐axis (B525‐H) represents GFP fluorescence intensity and the *y*‐axis (SSC‐H) represents relative particle size. Gate P1 indicates large fluorescent particles. Analyses were repeated twice.

To visualize SHH‐containing XLEVs, NIH3T3 fibroblasts were transduced with EGFP‐tagged SHH‐N (SHHN‐EGFP; Figure 2A–F). Large fluorescent vesicles (∼1 µm) were observed moving within the culture medium, particularly following CID treatment (Figure 2A,B). Quantification of vesicle density demonstrated a significant increase in SHH‐XLEVs upon CID stimulation (Figure 2C,D,I; Movie S1).

Pharmacological inhibition of Hsp90 (TAS‐116) or nSMase2 (GW4869) abolished CID‐induced SHH‐XLEV secretion (Figure 2E,F), indicating that both activities are required for this process. Consistently, expression of Rab18 nucleotide‐state mutants revealed opposing effects on SHH‐XLEV release. Cells expressing the GDP‐bound Rab18S22N mutant exhibited reduced intracellular SHH signal and increased extracellular SHH‐XLEV abundance, whereas the GTP‐bound Rab18Q67L mutant retained SHH intracellularly and markedly reduced XLEV release (Figure 2G,H,J; Movie S2). These findings indicate that Rab18 specifically promotes SHH‐XLEV biogenesis in its GDP‐bound state.

To directly assess XLEV size and SHH incorporation, conditioned media from piggyBac–SHHN‐mStayGold–transfected fibroblasts were analyzed by flow cytometry following CID stimulation. A population of large fluorescent particles (Gate P1) increased 2–3‐fold upon SHHN‐StayGold expression (Figure 2K–M), confirming that SHH is incorporated into XLEVs. Collectively, these data demonstrate that CID enriches Rab18‐GDP, thereby promoting SHH‐XLEV biogenesis in an Hsp90α‐ and nSMase2‐dependent manner.

### 3.3 SHH‐XLEVs are Secreted From the Perinuclear Region

**FIGURE 3:**
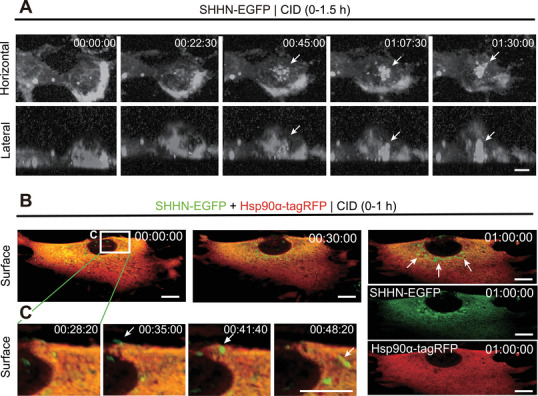
CID1067700 (CID) facilitates SHH‐XLEV secretion from the perinuclear region. (A) Three‐dimensional reconstructed time‐lapse spinning disk microscopy of SHHN‐EGFP in NIH3T3 cells coexpressing Hsp90α‐tagRFP following CID treatment (40 µM) for the indicated durations. Arrows indicate vertically moving SHH‐XLEV precursors. Time stamps indicate hours: minutes: seconds after stimulation. Scale bar, 10 µm. Related to Movie S3. (B, C) Time‐lapse lattice light‐sheet microscopy of SHHN‐EGFP– and Hsp90α‐tagRFP–coexpressing NIH3T3 cells following CID treatment (40 µM), shown at low (B) and high (C) magnification. Arrows indicate SHH‐XLEVs released from the perinuclear plasma membrane region. Scale bars, 10 µm. Related to Movie S4.

Time‐lapse imaging of SHHN‐EGFP–expressing fibroblasts during CID treatment revealed pronounced perinuclear accumulation of SHH signal within 30 min (Figure 3A; Movie S3). Vertical optical sections demonstrated progressive movement of SHH‐positive structures towards the plasma membrane from the perinuclear region within 1 h.

Lattice light‐sheet microscopy further showed that tubulovesicular SHH‐XLEVs (1–2 µm) were secreted exclusively from the perinuclear plasma membrane region (Figure 3B,C; Movie S4). Individual vesicles remained transiently associated with the cell surface before rapidly disengaging, supporting a spatially restricted mode of XLEV secretion centered on the perinuclear domain.

### 3.4 PI3K Signalling Recruits Rab18 into Perinuclear Endosomes

**FIGURE 4:**
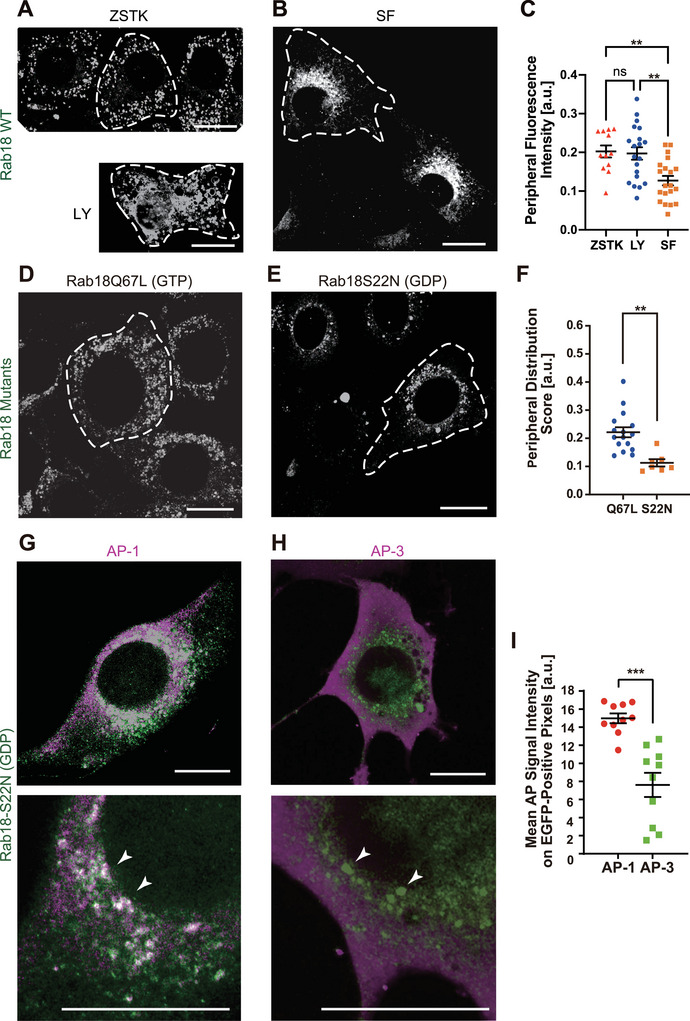
GDP‐bound Rab18 accumulates in perinuclear endosomes and promotes SHH‐XLEV secretion. (A–C) Airyscan microscopy of NIH3T3 cells stably expressing SHHN‐EGFP following treatment with ZSTK474 (1 µM, 1 h; A, upper), LY294002 (50 µM, 8 h; A, lower), or SF1670 (250 nM, overnight; B), with quantification of peripheral distribution scores (PDS; C). Scale bars, 20 µm. **p < 0.01; Welch's *t* test; *n* = 20. (D–F) Airyscan microscopy of NIH3T3 cells expressing EGFP‐Rab18Q67L (GTP‐bound mimetic; D) or EGFP‐Rab18S22N (GDP‐bound mimetic; E), with PDS quantification (F). Scale bars, 20 µm. **p < 0.01; Welch's *t* test; *n* = 7–16. (G–I) Airyscan immunofluorescence microscopy of EGFP‐Rab18S22N–expressing cells stained for AP‐1 (G) or AP‐3 (H) at low (upper) and high (lower) magnifications, with colocalization analysis (I). Arrowheads indicate perinuclear Rab18S22N puncta. Scale bars, 20 µm. ***p < 0.001; Welch's *t* test; n = 10.

To examine whether PI3K signalling regulates the subcellular distribution of Rab18, fibroblasts stably expressing Rab18‐EGFP were treated with PI3K inhibitors (ZSTK474 or LY294002) or with the PI3K agonist/PTEN antagonist SF1670 (Figure 4A–C). Inhibition of PI3K signalling resulted in enlargement and dispersion of Rab18‐positive vesicles throughout the cytoplasm (Figure 4A). In contrast, SF1670 treatment induced pronounced perinuclear clustering of Rab18‐positive vesicles (Figure 4B).

Quantitative analysis revealed a significant reduction in Rab18‐EGFP fluorescence intensity at the cell periphery following SF1670 treatment compared with LY294002 treatment (Figure 4C), indicating that PI3K signalling promotes Rab18 accumulation in the perinuclear region.

To determine whether this redistribution reflects enrichment of a specific Rab18 nucleotide state, we compared the localization of GDP‐ and GTP‐bound Rab18 mimetics. The Rab18S22N (GDP‐bound) mutant showed significantly stronger perinuclear accumulation than the Rab18Q67L (GTP‐bound) mutant (Figure 4D–F). Notably, cells expressing Rab18S22N also tended to exhibit a flatter and more laterally expanded morphology compared with Rab18Q67L‐expressing cells, although this effect was not quantitatively assessed. The apparent increase in puncta size observed with Rab18Q67L expression is consistent with homotypic vesicle fusion previously reported for other GTP‐bound Rab proteins (Nielsen et al. 1999).

To identify the perinuclear compartments containing Rab18‐GDP, Rab18S22N‐EGFP–expressing cells were co‐stained for the adaptor proteins AP‐1 and AP‐3 (Figure 4G–I). Rab18‐GDP showed stronger colocalization with AP‐1 than with AP‐3, consistent with localization to endosomal compartments involved in TGN‐to‐endosome trafficking rather than lysosomal pathways. Our preliminary observation also suggested that those particles did not colocalize to cis‐Golgi marker GM130. Together, these data indicate that PI3K signalling promotes enrichment of Rab18‐GDP within perinuclear endosomal compartments that serve as precursors for SHH‐XLEV biogenesis.

### 3.5 Rab18‐GDP Endosomes Recruit Hsp90α

**FIGURE 5:**
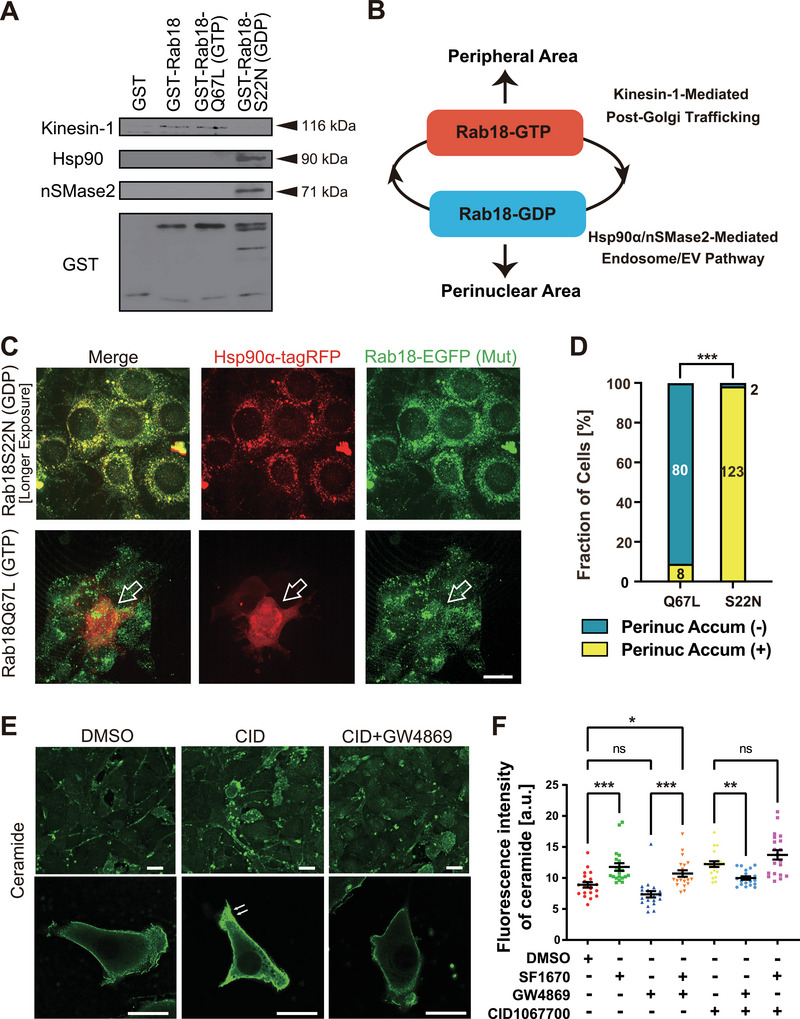
Hsp90α and nSMase2 are effectors of GDP‐bound Rab18. (A) GST pull‐down assays using mouse brain lysates incubated with GST‐tagged Rab18 mutants, immunoblotted with the indicated antibodies. Rab18WT and Rab18Q67L preferentially associated with kinesin‐1 (KIF5A), whereas Rab18S22N preferentially bound Hsp90α and nSMase2. (B) Schematic summary of Rab18‐GTP– and Rab18‐GDP–specific effector interactions. (C, D) Spinning disk microscopy of NIH3T3 cells co‐expressing Hsp90α‐tagRFP and EGFP‐Rab18 mutants (C), with quantification of cells showing perinuclear Hsp90α accumulation (D). Open arrows, diffuse cytoplasmic distribution of Hsp90α in a Rab18‐GTP‐expressing cell. Scale bar, 20 µm. ***p < 0.001; chi‐square test; *n* = 88–125 cells. (E, F) Ceramide immunocytochemistry of NIH3T3 cells treated with DMSO, CID (40 µM), or CID plus GW4869 (1.25 µM, 24 h), shown at low and high magnification (E), with quantification (F). Arrows indicate peripheral ceramide accumulation. Scale bars, 20 µm. *p < 0.05; **p < 0.01; ***p < 0.001; one‐way ANOVA; *n* = 20.

To investigate how Rab18‐GDP facilitates SHH‐XLEV maturation, we performed GST pull‐down assays using GDP‐ or GTP‐bound Rab18 fusion proteins incubated with mouse brain lysates (Figure 5A). Rab18‐GDP preferentially associated with Hsp90α and nSMase2, whereas Rab18‐GTP selectively bound the kinesin‐1 motor protein KIF5. These binding preferences are consistent with previous reports implicating Rab18 in kinesin‐dependent organelle transport (Guadagno et al. 2020).

Because kinesin and cytoplasmic dynein generate opposing forces that determine organelle positioning, loss of kinesin‐1 association upon Rab18‐GDP enrichment is expected to favour perinuclear accumulation of Rab18‐associated membranes (Tanaka et al. 1998; Ueno et al. 2011). These nucleotide‐state–dependent interactions provide a molecular basis for site‐specific maturation of SHH‐XLEV precursors in the perinuclear region (Figure 5B).

To assess whether Rab18 nucleotide status influences Hsp90α localization in cells, Hsp90α‐tagRFP was co‐expressed with Rab18S22N‐EGFP or Rab18Q67L‐EGFP (Figure 5C,D). Hsp90α strongly colocalized with Rab18‐GDP in perinuclear compartments, whereas in Rab18‐GTP–expressing cells, Hsp90α was diffusely distributed throughout the cytoplasm with minimal punctate overlap. These results indicate that Rab18‐GDP recruits Hsp90α to perinuclear endosomes.

### 3.6 Rab18‐GDP Enhances Ceramide Production via nSMase2

Because nSMase2 is a membrane‐associated enzyme involved in ceramide biosynthesis (Piacentino et al. 2022), we examined whether Rab18‐GDP association influences cellular ceramide levels. Immunocytochemical analysis revealed that CID treatment significantly increased ceramide accumulation, particularly at the cell periphery (Figure 5E,F).

This increase was abolished by co‐treatment with the nSMase2 inhibitor GW4869 and restored by additional SF1670 treatment. Given that ceramide promotes endosomal enlargement and EV biogenesis (Fiorani et al. 2023; Horbay et al. 2022; Li et al. 1999), these data suggest that Rab18‐GDP enhances SHH‐XLEV secretion in part by facilitating nSMase2‐dependent ceramide production.

### 3.7 Rab18 Nucleotide State Modulates SHH Secretion Modalities

**FIGURE 6:**
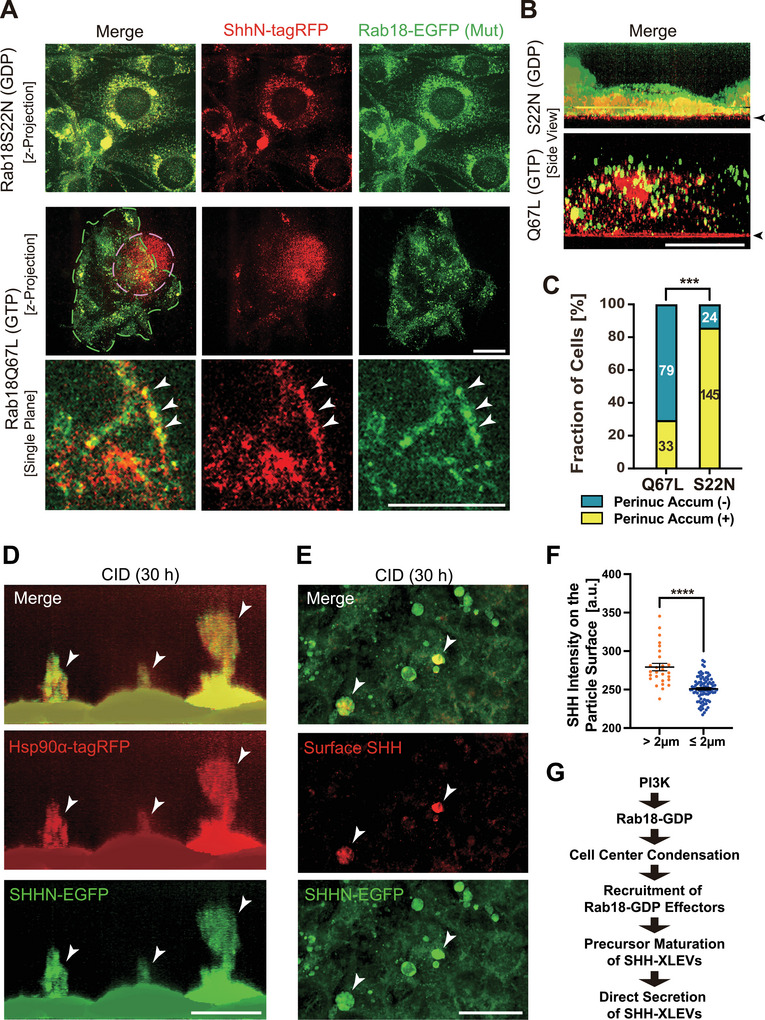
GDP‐bound Rab18 promotes perinuclear SHH‐XLEV secretion. (A–C) Spinning disk microscopy of NIH3T3 cells coexpressing SHHN‐tagRFP and EGFP‐Rab18 mutants, shown as z‐projections (A), single planes (A), and side views (B), with quantification of perinuclear SHH accumulation (C). Dotted lines, periphery of the Rab18‐GTP‐expressing colony (green) and basolateral SHH deposition (magenta). Arrowheads, colocalization spots of Rab18‐GTP and SHH on the plasma membrane (A) and Rab18‐GTP‐specific basolateral SHH deposition (B). Scale bars, 20 µm. ***p < 0.001; chi‐square test; *n* = 88–125 cells. Related to Movie S5. (D) Spinning disk microscopy of NIH3T3 cells undergoing burst‐like secretion of SHHN‐EGFP and Hsp90α‐tagRFP following prolonged CID treatment (arrowheads, 30 h). Scale bar, 20 µm. Related to Figure S3 and Movie S6. (E, F) Three‐dimensional reconstruction of surface SHH immunofluorescence (red) in SHHN‐EGFP–expressing NIH3T3 cells (E), with quantification of SHH signal intensity appearing on the particle surface (E, arrowheads; F). Scale bar, 20 µm. ****p < 0.0001; Welch's t test; *n* = 27 versus 90 cells. (G) Working model of the PI3K–Rab18‐GDP–dependent SHH‐XLEV secretion pathway. Related to Figure S4.

To examine how Rab18 nucleotide status influences SHH trafficking, NIH3T3 cells were co‐transduced with SHHN‐tagRFP and Rab18S22N‐ or Rab18Q67L‐EGFP (Figure 6A–C). As expected, Rab18‐GDP promoted strong perinuclear accumulation of SHHN‐tagRFP. In contrast, Rab18‐GTP–positive vesicles were dispersed throughout the cytoplasm and partially colocalized with SHHN signals at the plasma membrane.

Notably, Rab18‐GTP expression was associated with deposition of SHH‐N onto large extracellular foci (approximately 50–100 µm) on the coverslip surface beneath and adjacent to cell colonies (Figure 6A,B; Movie S5). These structures are consistent with ECM‐associated SHH assemblies previously implicated in canonical hedgehog signalling. These observations indicate that distinct Rab18 nucleotide states differentially influence the spatial mode of SHH secretion.

### 3.8 Prolonged CID Treatment Induces Extracellular Hsp90α‐Rich SHH‐XLEV Assemblies

Following extended CID treatment (24–30 h), large extracellular Hsp90α‐enriched condensations (20–40 µm in diameter) containing SHH‐XLEVs were frequently observed (Figure 6D; Figure S3A–F; Movie S6). These structures were often connected to the cell surface by cytoneme‐like processes, and their abundance was significantly increased by CID treatment (Figure S3G).

Airyscan microscopy revealed that SHH‐N localized primarily to discrete punctate structures embedded within a surrounding matrix of Hsp90α‐dominant material (Figure S3H,I). Similar extracellular EV‐associated fibrous structures have been described in developing mouse embryos (Tanaka et al. 2024), supporting the idea that SHH‐XLEVs can participate in large‐scale extracellular assemblies.

### 3.9 SHH‐XLEV Secretion Involves Vesicle Surface Remodeling

Detergent‐free immunofluorescence microscopy of SHHN‐EGFP–expressing fibroblasts following prolonged CID treatment revealed round SHH‐positive particles of varying sizes near the cell surface (Figure 6E,F). Quantitative analysis demonstrated a significant positive correlation between SHH signal intensity and vesicle diameter (Figure 6F), suggesting dynamic remodeling of SHH‐XLEVs upon secretion. These observations are consistent with vesicle surface rearrangements during extracellular release.

### 3.10 SHH‐XLEV–Containing Secretomes Exhibit High Angiogenic Activity

**FIGURE 7:**
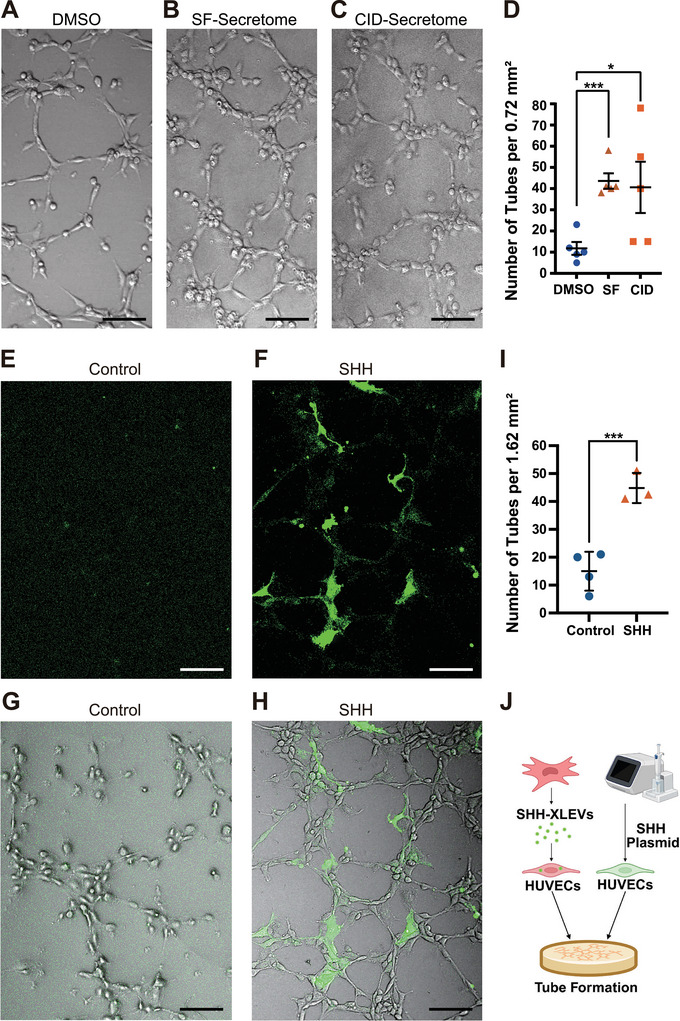
SHH‐XLEVs exhibit high angiogenic activity. (A–D) Bright‐field images of human umbilical vein endothelial cells (HUVECs) plated on Matrigel in the presence of secretomes derived from hMSCs treated with DMSO (A), SF1670 (250 nM; B), or CID (40 µM; C), with quantification of tube formation (D). Scale bars, 100 µm. *p < 0.05; ***p < 0.001; one‐way ANOVA; *n* = 5 independent experiments. (E–I) Fluorescence (E,F) and bright‐field‐merged microscopy (G,H) of control (E,G) or SHHN‐StayGold–transfected (F,H) HUVECs, with quantification of tube formation (I). Scale bars, 100 µm. *p < 0.05; ***p < 0.001; one‐way ANOVA; *n* = 3–4 independent experiments. (J) Schematic summary illustrating that SHH contributes to the angiogenic activity of XLEVs in recipient endothelial cells.

To assess the functional relevance of SHH‐XLEVs, we examined the angiogenic activity of secretomes using an established HUVEC tube formation assay (Figure 7A–C). HUVECs mixed with SF‐ or CID‐induced hMSC secretomes formed vessel‐like networks at levels approximately 4–5‐fold higher than those observed with DMSO‐induced secretomes (Figure 7D), indicating robust pro‐angiogenic activity.

To determine whether SHH contributes directly to this activity, HUVECs were transfected with an SHHN‐StayGold expression vector and subjected to the same assay (Figure 7E,F). SHHN‐expressing HUVEC cultures exhibited approximately fourfold higher tube formation compared with control cultures (Figure 7G), supporting a functional role for SHH in mediating the angiogenic effects associated with SHH‐XLEVs (Figure 7H).

## 4 Discussion

In this study, we characterized angiogenic SHH‐XLEVs as a population of large extracellular vesicles whose secretion is selectively enhanced through a PI3K–Rab18‐dependent pathway (Figure 6G; Figure S4). Rather than emphasizing vesicle purity, our analyses establish the existence, enrichment, and regulated secretion of SHH‐XLEVs, which are molecularly and spatially distinct from canonical small EV populations. The strong angiogenic activity of SHH‐XLEVs (Figure 7) suggests that they represent a previously underappreciated EV subpopulation involved in PI3K‐mediated developmental and regenerative angiogenic signalling pathways (Kobialka et al. 2023; Ma et al. 2009).

A notable finding of this study is that pharmacological perturbation of Rab activity selectively enhances SHH‐XLEV secretion. Treatment with the pan‐Rab inhibitor CID1067700, which enriches GDP‐bound Rab species, robustly increased SHH‐XLEV release while tending to suppress small EV secretion (Figure 1A; Figure S1). Although CID can inhibit multiple Rab proteins, several lines of evidence support a dominant role for Rab18 in this process. Rab18 is a major constituent of SHH‐XLEV precursors, as shown by our data and previous reports (Coulter et al. 2018; Wang et al. 2022), and manipulation of Rab18 nucleotide status exerted bidirectional effects on SHH‐XLEV secretion, with GDP‐locked and GTP‐locked mutants enhancing and suppressing release, respectively (Figure 2G–J). These observations place Rab18 in an unusual regulatory position within the EV secretion landscape, where GDP‐bound Rab activity promotes the release of large EVs.

Mechanistically, our data suggest that Rab18 integrates spatial organization and enzymatic regulation during SHH‐XLEV biogenesis. At the level of subcellular geometry, Rab18‐GDP‐positive vesicles preferentially accumulated in AP‐1‐positive perinuclear endosomes, whereas Rab18‐GTP was more broadly distributed throughout the cytoplasm (Figure 4D–F). Kinesin‐1 exhibited a strong binding preference for GTP‐bound Rab18 (Figure 5A,B), consistent with the GTP‐dependent engagement of kinesin motors by Rab GTPases reported for other Rab–kinesin interactions (Niwa et al. 2008; Ueno et al. 2011). These findings support a model in which Rab18‐GDP vesicles are biased towards centripetal transport, likely mediated by cytoplasmic dynein, resulting in perinuclear clustering through an altered motor tug‐of‐war balance.

PI3K signalling emerged as an upstream regulator of this Rab18‐dependent process. PI3K stimulation significantly enhanced SHH‐XLEV secretion (Figure 1A), consistent with previous observations (Wang et al. 2022), and shifted Rab18‐EGFP localization towards the perinuclear region. This suggests that PI3K signalling promotes the generation or stabilization of GDP‐bound Rab18 on SHH‐XLEV precursors. A similar PI3K–Rab18 relationship has been reported in adipocytes, where insulin‐mediated PI3K signalling recruits Rab18 to lipid droplets (Pulido et al. 2011).

At the level of enzymatic regulation, Rab18‐GDP was preferentially associated with Hsp90α and nSMase2 (Figure 5A), both of which were required for efficient CID‐stimulated SHH‐XLEV secretion based on pharmacological and genetic evidence (Figure 2A–J). Hsp90α localized to Rab18‐GDP‐positive perinuclear endosomes (Figure 6A,C) and was co‐secreted with SHH‐XLEVs, particularly at later stages (Figure 6D; Figure S3). This unconventional secretion of Hsp90α may provide new insights into previously described Hsp90α‐dependent mechanisms of extracellular antigen presentation and immune modulation (Srivastava 2002). nSMase2, which converts sphingomyelin into the bioactive lipid ceramide (Quadri and Bieberich 2025), has been implicated in EV biogenesis (Fiorani et al. 2023; Horbay et al. 2022). Consistently, CID treatment significantly elevated cellular ceramide levels (Figure 5E,F), suggesting that Rab18‐GDP‐associated nSMase2 activity contributes to local membrane remodeling that favours SHH‐XLEV formation.

The secretion route of SHH‐XLEVs is distinct from that of canonical small EVs enriched in tetraspanins such as CD81 and CD9, whose punctate distribution is dispersed throughout the cytoplasm (Mathieu et al. 2021). While small EV secretion depends on both GDP‐bound Rab7 (Jiang et al. 2025) and GTP‐bound Arl13b/Rab27a (Ostrowski et al. 2010; Verweij et al. 2022), SHH‐XLEV secretion appears to rely on pronounced perinuclear accumulation followed by slow, large‐scale membrane extension. This latter step may substitute for long‐range microtubule‐based transport and instead depend on actomyosin‐driven mechanical forces (Illukkumbura et al. 2020; Murrell et al. 2015), consistent with the direct vertical elongation of tubulovesicular SHH‐XLEV precursors observed in our study (Figure 3A; Movie S3).

Previous studies from our group and others have shown that increased SHH‐N loading enhances EV size and signalling potency (Coulter et al. 2018; Mackie et al. 2012; Tanaka et al. 2005; Wang et al. 2022). This effect may be partly attributable to lipid and cholesterol modifications of SHH‐N that increase its affinity for lipid bilayers (Lewis et al. 2001; Pepinsky et al. 1998). In addition, homophilic interactions among SHH molecules (Whalen et al. 2013) may facilitate fusion among SHH‐associated intraluminal vesicles within multivesicular bodies, thereby promoting the formation of extra‐large vesicular structures.

In summary, we propose a PI3K–Rab18‐GDP‐dependent mechanism that selectively enhances the unconventional secretion of SHH‐XLEVs. This regulatory logic, in which GDP‐bound Rab18 promotes large EV release, contrasts with prevailing models of EV secretion that emphasize GTP‐bound Rab activity (Blanc and Vidal 2018). By revealing an alternative pathway for the controlled secretion of large bioactive vesicles, our findings broaden current models of EV biogenesis and provide a conceptual framework for future investigations into XLEV biology and their roles in angiogenic and regenerative signalling contexts.

## Author Contributions

**Shuo Wang**: conceptualization, methodology, software, data curation, investigation, validation, writing – review and editing, writing – original draft, visualization. **Rio Imai**: methodology, software, data curation, investigation, visualization, writing – review and editing. **Yuya Kaneko**: investigation, methodology, visualization, writing – review and editing, data curation. **Yosuke Tanaka**: conceptualization, methodology, investigation, validation, supervision, funding acquisition, visualization, project administration, resources, writing – original draft, writing – review and editing.

## Conflicts of Interest

Y.T. and S.W. are inventors on patent applications related to this work, including a Japanese patent application (No. 2022–078750), a U.S. patent application (No. 18/864610), and a European patent application (No. 23803594.3). The other authors declare no competing interests.

## Supporting information



Supplementary Information: jex270112‐sup‐0001‐SuppMat.pdf

Supplementary Information: jex270112‐sup‐0002‐MovieS1_wang.mp4

Supplementary Information: jex270112‐sup‐0003‐MovieS2_wang.mp4

Supplementary Information: jex270112‐sup‐0004‐MovieS3_wang.mp4

Supplementary Information: jex270112‐sup‐0005‐MovieS4_wang.mp4

Supplementary Information: jex270112‐sup‐0006‐MovieS5_wang.mp4

Supplementary Information: jex270112‐sup‐0007‐MovieS6_wang.mp4

## Data Availability

The data that support the findings of this study are available from the corresponding author upon reasonable request.
