# Supplementary material for: Unconventional Secretion of Angiogenic Sonic Hedgehog–Containing Extra‐Large Extracellular Vesicles is Driven by PI3K–Rab18‐GDP Signalling
Source: J Extracell Biol. 2026 Jan 28;5(2):e70112. doi: 10.1002/jex2.70112 (PMC12848524; doi:10.1002/jex2.70112)
Supplement: Supplementary file 1 — Supplementary Information: jex270112‐sup‐0001‐SuppMat.pdf [file JEX2-5-e70112-s003.pdf]

Supplementary Information

Unconventional secretion of angiogenic sonic hedgehog–containing extra-large extracellular vesicles is driven by PI3K–Rab18-GDP signalling

Shuo Wang, Rio Imai, Yuya Kaneko, Yosuke Tanaka*

Laboratory of Molecular Cell Dynamics/Cytoarchitectonics, Department of Cell Biology and Anatomy, Graduate School of Medicine, The University of Tokyo, Hongo, Tokyo, 113-0033, Japan.

Supplementary Figures 1–4
Supplementary Movie Legends 1–6

SUPPLEMENTARY FIGURES

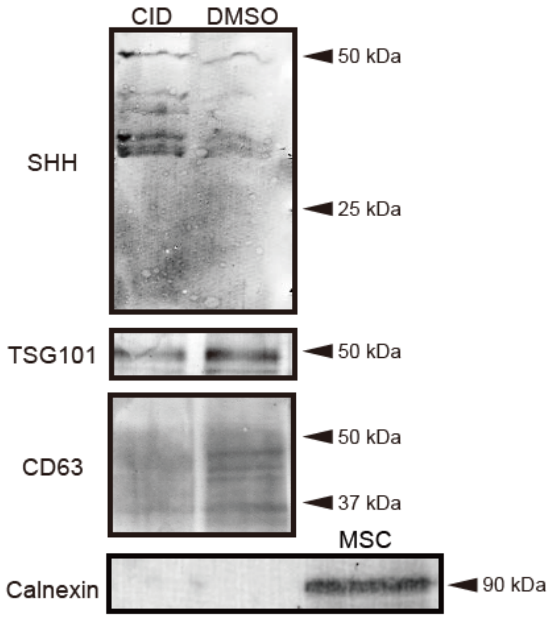

Supplementary Figure S1. Short-term CID treatment enriches SHH-positive EVs while reducing canonical sEV markers

Immunoblot analysis of EVs collected from hTERT-MSC–conditioned media after 2 h treatment with CID1067700 (CID) and DMSO, showing induction of SHH-positive EV secretion with reduced levels of canonical sEV markers (TSG101 and CD63). ER-derived contaminants (calnexin) are below the detection level, compared with the same amount of cell lysates (MSC). Related to **Fig. 1A**.

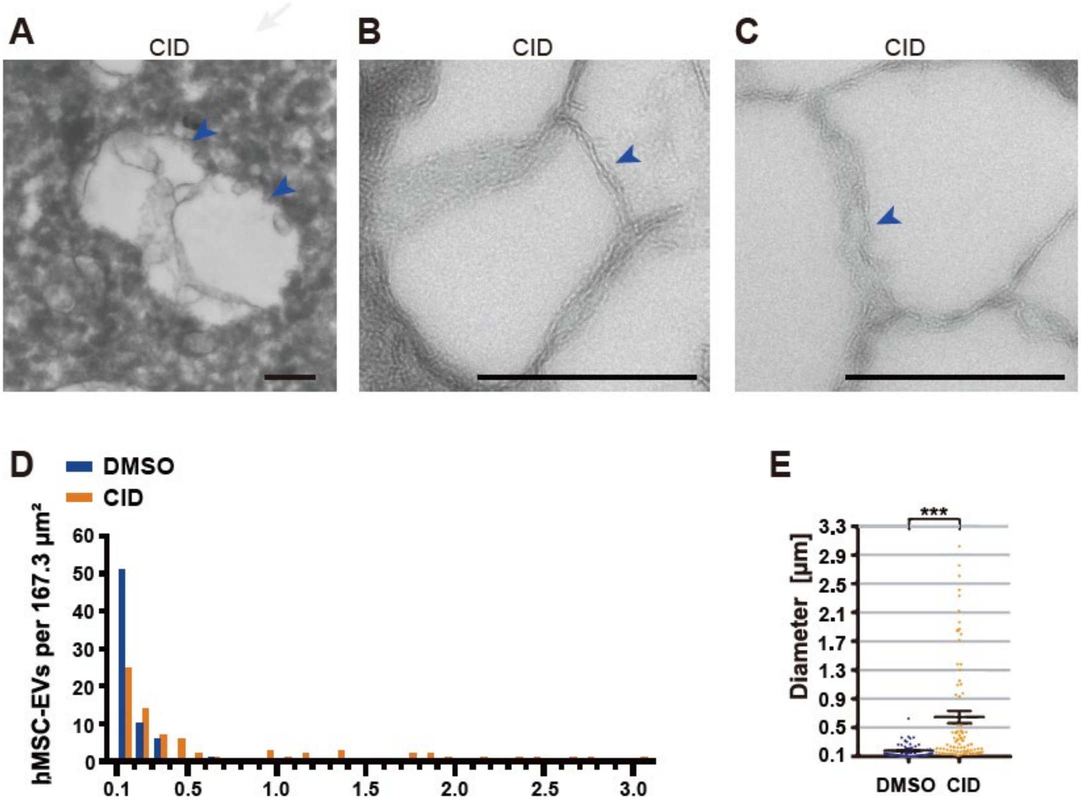

Supplementary Figure S2. TEM-based morphological validation and size quantification of CID-induced XLEVs

(A) Representative TEM images of EV pellets prepared from CID-stimulated bone-marrow-hMSC conditioned media (conditions as in **Fig. 1E**). Arrowheads, XLEVs. Scale bar, 500 nm.

(B, C) Higher-magnification TEM images highlighting membrane-limited large vesicular structures consistent with XLEVs (arrowheads), distinguishing them from non-vesicular particles. Scale bars, 500 nm.

(D, E) Quantification of vesicle size distributions from TEM images, demonstrating a significant increase in XLEV abundance/size upon CID treatment. ***$p < 0.0001$, Welch's t test, n = 68–75. Related to **Fig. 1E,F**.

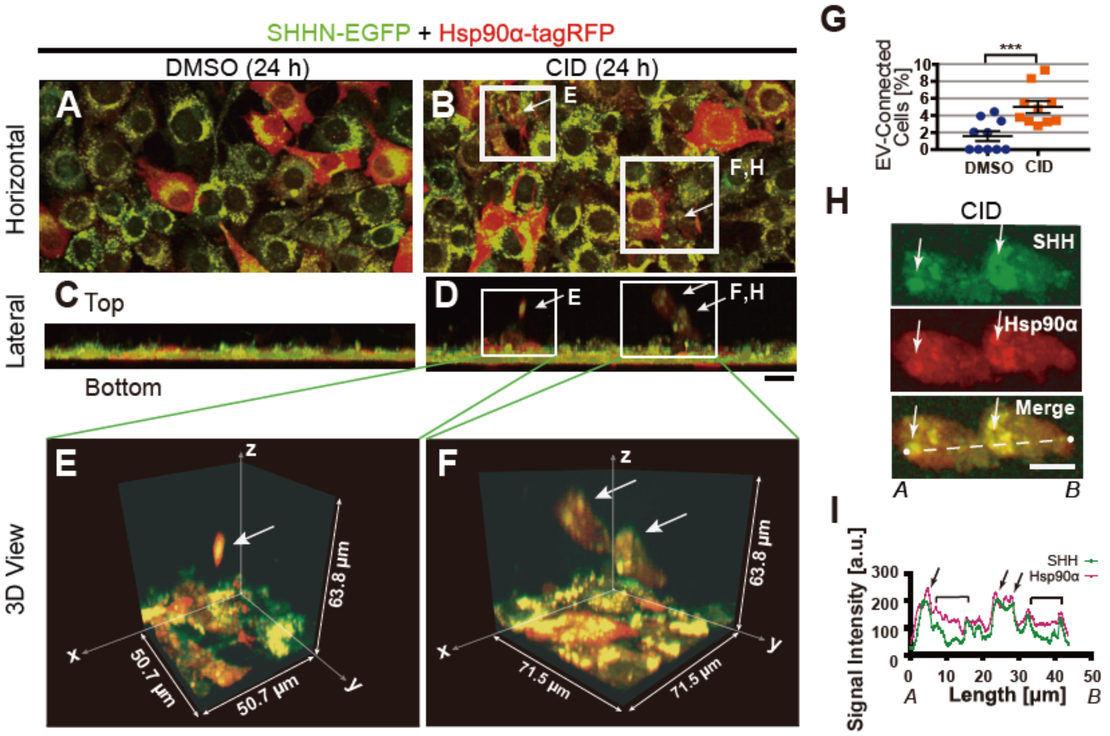

Supplementary Figure S3. Long-term CID treatment induces extracellular Hsp90α-rich condensates associated with SHH-XLEVs

(A–F) Representative fluorescence images showing large extracellular Hsp90α-enriched condensates associated with SHH-positive vesicular structures following prolonged CID treatment. Scale bar, 20 µm.

(G) Quantification of the number of SHH-containing extracellular condensates, showing a CID-dependent increase. ***$p < 0.0001$, Welch's t test, n = 10.

(H, I) Airyscan imaging revealing punctate SHH signals embedded within surrounding Hsp90α-dominant amorphous material. Scale bar, 20 µm. Related to **Fig. 6D and Movie S6.**

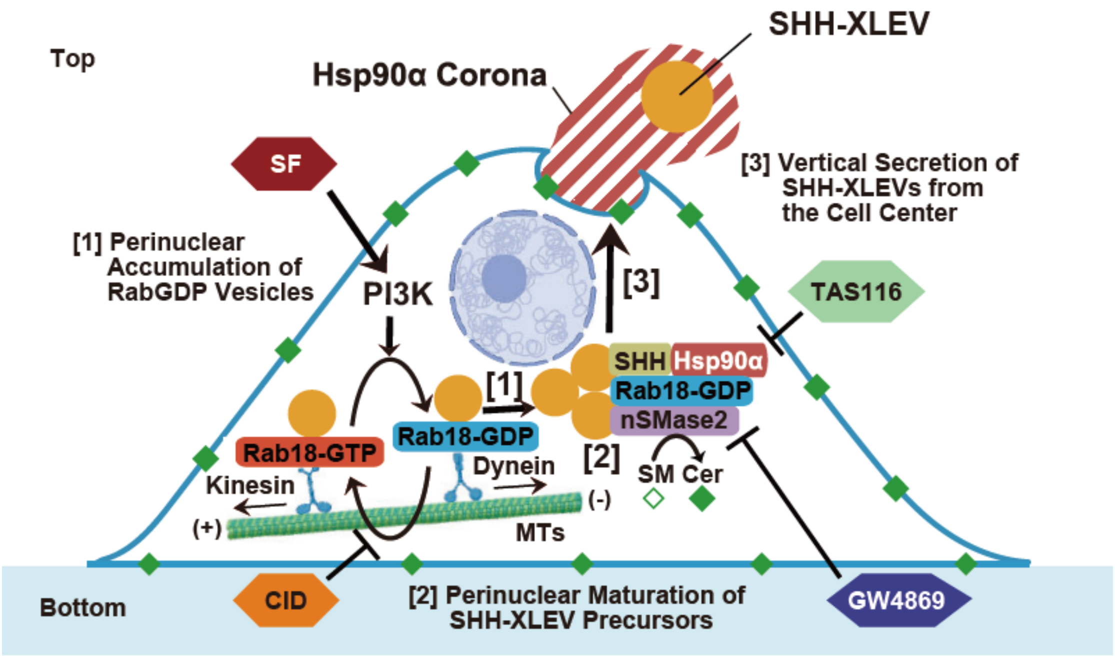

Supplementary Figure S4. PI3K–Rab18-GDP pathway framework for SHH-XLEV secretion

Schematic model summarizing the proposed PI3K–Rab18-GDP–dependent pathway for SHH-XLEV biogenesis and polarized secretion, highlighting Rab18 nucleotide-state regulation, recruitment of Hsp90α and nSMase2, and perinuclear release. Related to **Fig. 6G.**

SUPPLEMENTARY MOVIE LEGENDS

Movie S1. CID1067700 (CID) induces secretion of sonic hedgehog–containing extra-large extracellular vesicles (SHH-XLEVs)

Time-lapse spinning disk fluorescence microscopy of NIH3T3 cells transduced with an SHHN-EGFP expression vector under the indicated pharmacological conditions. CID treatment (40 µM, 24 h) markedly enhanced SHH-XLEV secretion into the culture medium, whereas this effect was suppressed by co-treatment with the Hsp90 inhibitor TAS-116 (0.5 µM, 48 h) or the nSMase2 inhibitor GW4869 (1.25 µM, 24 h). The movie spans 100 s. Scale bar, 50 µm. Related to **Fig. 2A–F and Fig. 2I**.

Movie S2. GDP-bound Rab18 promotes SHH-XLEV secretion

Time-lapse spinning disk fluorescence microscopy of NIH3T3 cells co-transduced with SHHN-tagRFP and EGFP-tagged Rab18 nucleotide-state mutants (S22N, GDP-bound; Q67L, GTP-bound). Images of the red channel are shown. SHH-XLEV secretion was enhanced in cells expressing Rab18-GDP, whereas SHH predominantly accumulated intracellularly in cells expressing Rab18-GTP. The movie spans 100 s. Scale bar, 50 µm. Related to **Fig. 2G,H,J.**

Movie S3. CID1067700 (CID) induces biphasic perinuclear SHH vesicle dynamics

Time-lapse spinning disk fluorescence microscopy of NIH3T3 cells co-transduced with SHHN-EGFP and Hsp90α-tagRFP. Green-channel images are shown. SHHN-EGFP–positive membrane organelles first accumulate horizontally in the perinuclear region (arrow) and subsequently move vertically toward the plasma membrane. The movie spans 1.5 h. Scale bar, 20 µm. Related to **Fig. 3A**.

Movie S4. CID1067700 (CID) induces SHH-XLEV secretion from the perinuclear region

Time-lapse lattice light-sheet microscopy of the cell surface of an NIH3T3 cell co-transduced with SHHN-EGFP (green) and Hsp90α-tagRFP (red). Both channels are shown. A green SHH-XLEV (1–2 µm in diameter) is secreted from the perinuclear region. The movie spans 1 h. Scale bar, 10 µm. Related to **Fig. 3B,C**.

Movie S5. GDP- and GTP-bound Rab18 differentially regulate SHH distribution

in NIH3T3 cells

Rotation views of three-dimensional projection of fluorescence microscopy images of NIH3T3 cells expressing GFP-fused Rab18 nucleotide-state mutants—dominant-negative Rab18S22N (GDP-bound) or constitutively active Rab18Q67L (GTP-bound) (green)—and transduced with SHH-tagRFP (red). Images were acquired using a 100× objective lens and reconstructed as 3D projections using Fiji software. Scale bar, 20 μm. Related to **Fig. 6B**.

Movie S6. Long-term CID1067700 (CID) treatment induces extracellular Hsp90α-rich condensates containing SHH-XLEVs

Rotational view of three-dimensional reconstructed z-stack images of NIH3T3 cells co-transduced with SHHN-EGFP (green) and Hsp90α-tagRFP (red), showing large extracellular condensates positioned above the cell layer and associated with SHH-XLEVs. Scale bar, 30 μm. Related to **Fig. 6D and Supplementary Fig. S3**.
